# 2D, 3D-QSAR and docking studies of 1,2,3-thiadiazole thioacetanilides analogues as potent HIV-1 non-nucleoside reverse transcriptase inhibitors

**DOI:** 10.1186/2191-2858-2-22

**Published:** 2012-06-12

**Authors:** Shailesh V Jain, Manjunath Ghate, Kamlendra S Bhadoriya, Sanjaykumar B Bari, Amar Chaudhari, Jayshri S Borse

**Affiliations:** 1 Institute of pharmacy, Nirma University, Ahmedabad, Gujarat, 382481, India; 2 R. C. Patel Institute of Pharmaceutical Education and Research, Shirpur, Dhule District, Maharashtra, 425405, India

**Keywords:** 1,2,3-Thiadiazole thioacetanilides, QSAR, Docking, HIV-RT inhibitors;*k*-nearest neighbor molecular field analysis (kNN-MFA)

## Abstract

**Background:**

The discovery of clinically relevant inhibitors of HIV-RT for antiviral therapy has proven to be a challenging task. To identify novel and potent HIV-RT inhibitors, the quantitative structure–activity relationship (QSAR) approach became very useful and largely widespread technique forligand-based drug design.

**Methods:**

We perform the two- and three-dimensional (2D and 3D) QSAR studies of a series of 1,2,3-thiadiazole thioacetanilides analogues to elucidate the structural properties required for HIV-RT inhibitory activity.

**Results:**

The 2D-QSAR studies were performed using multiple linear regression method, giving *r*^2^ = 0.97 and *q*^2^ = 0.94. The 3D-QSAR studies were performed using the stepwise variable selection *k*-nearest neighbor molecular field analysis approach; a leave-one-out cross-validated correlation coefficient *q*^2^ = 0.89 and a non-cross-validated correlation coefficient *r*^2^ = 0.97 were obtained. Docking analysis suggests that the new series have comparable binding affinity with the standard compounds.

**Conclusions:**

This approach showed that hydrophobic and electrostatic effects dominantly determine binding affinities which will further useful for development of new NNRTIs.

## Background

Acquired immunodeficiency syndrome (AIDS), caused by human immunodeficiency virus (HIV) infection, is still one of the most important challenges for the chemotherapy of the early 21st century [1]. The status of the HIV/AIDS epidemic has evolved with time and by many estimates the global adult prevalence of HIV has stabilized at slightly less than 1% of the world population [2]. The number of people living with HIV worldwide continued to grow in 2010, reaching an estimated 33.4 million. The total number of people living with the virus in 2010 was more than 20% higher than the number in 2000, and the prevalence was roughly threefold higher than in 1990. There are about 2.7 million adult and children are newly infected by HIV and about 2 million are died by AIDS [3].

HIV reverse transcriptase (RT) is a DNA polymerase enzyme that converts single-stranded viral genomic RNA into double-stranded DNA. HIV RT is one of the three key enzymes in the HIV life cycle and the primary target of numerous anti-viral drug discovery efforts [4]. Due to the essential role of the HIV RT in the viral replication, the inhibition of RT is regarded as one of the most attractive targets in the anti-HIV chemotherapy [5]. Current anti-AIDS therapy is based on drugs that belong either to the class of nucleoside/nucleotide (NRTIs/NtRTIs) and non-nucleoside reverse transcriptase inhibitors (NNRTIs), protease, or entry inhibitors. NNRTIs are a structurally diverse group of compounds which interact with a specific allosteric non-substrate binding pocket site of HIV-1 RT (non-nucleoside inhibitor binding pocket), leading to a non-competitive inhibition of the enzyme [6]. Association of an NNRTI with the reverse transcriptase is thought to inhibit chain elongation by altering the motions of the protein residues that interact with the nucleic acid chain [7]. NNRTIs are an important part of the successful combination therapy for HIV-1 known as highly active anti-retroviral therapy [8].

Among the structurally diverse HIV-1 NNRTIs, substituted azole-thioacetanilide scaffolds have been of much interest for the development of novel NNRTIs because of their high potency and low toxicity against HIV-1 wild-type and resistant strains. It is worth noting that halogenated phenyl rings were regarded as ubiquitous moieties in numerous NNRTI molecules, and the substitution pattern of halogens has a significant influence on the overall protein–ligand binding affinity and anti-HIV activity [9].

The quantitative structure–activity relationship (QSAR) approach became very useful and largely widespread for the prediction of biological activities, particularly in drug design. This approach is based on the assumption that the variations in the properties of the compounds can be correlated with changes in their molecular features [10].

Thisstudy aimed to elucidate the structural features of 1,2,3-thiadiazole thioacetanilides derivatives required for HIV-RT inhibition and to obtain predictive two- and three-dimensional QSAR (2D- and 3D-QSAR) models. Further, the binding mode of the active molecule with the active site amino acid residues of reverse transcriptase enzyme was performed by docking using Glide XP. It is expected that such 3D-QSARs and ligand–receptor-based molecular docking studies of 1,2,3-thiadiazole thioacetanilides derivatives will provide better tools for rational design of promising HIV-1 NNRT inhibitors having greater therapeutic safety and efficacy.

### Experimental methods

All molecular modeling studies (2D and 3D) were performed using the Molecular Design Suite (VLife MDS software package, version 3.5; from VLife Sciences, Pune, India) [11]. Docking studies were carried out using Schrodinger molecular modeling user interface implemented on Acer PC with a Pentium IV processor and Windows XP operating system. The structures of all compounds were sketches in Chem sketch version 12.0 [12]. All structures are cleaned and 3D optimized. Energy minimization and geometric optimization were conducted using the Merck molecular force field method with the root mean squaregradient set to 0.01 kcal/molÅ, and the iteration limit to 10,000. The conformers for all structures are generated and selected the low energy conformer for each compound and used for further study.

### 2D-QSAR study

#### Dataset and molecular modeling for 2D-QSAR

The effective concentration data on 1,2,3-thiadiazole thioacetanilides derivatives (Table 1) were taken from the literature [13]. The total set of compounds was divided into a training set (12 compounds) for generating 2D-QSAR models and a test set (10 compounds) for validating the quality of the models. Selection of molecules in the training set and test is a key and important feature of any QSAR model. Therefore, the care was taken in such a way that biological activities of all compounds in test lie within the maximum and minimum value range of biological activities of training set of compounds. The Uni-Column Statistics of test and training sets further reflected the correct selection of test and training sets. A Uni-Column statistics for training set and test set were generated to check correctness of selection criteria for trainings and test set molecules (Table 2).

**Table 1:** Molecular structures of compounds and their NNRT inhibitory activity

**ID**	***R***^**1**^	***R***^**2**^	***X***	***Y***	**EC**_**50**_**(μM)**	**pEC**_**50**_
7a1	H	H	Cl	H	26.11	4.58
7a4	H	H	Br	H	22.42	4.64
7a5^a^	H	H	Br	CH_3_	13.73	4.86
7a6^a^	H	H	Br	CO_2_C_2_H_5_	5.21	5.28
7a7	H	H	Br	CO_2_CH_3_	5.1	5.29
7b1^a^	H	OMe	Cl	H	2.63	5.58
7b2	H	OMe	NO_2_	H	0.99	6
7b4^a^	H	OMe	Br	H	3.19	5.49
7b5^a^	H	OMe	Br	CH_3_	5.06	5.29
7b6	H	OMe	Br	CO_2_C_2_H_5_	3.01	5.52
7b7^a^	H	OMe	Br	CO_2_CH_3_	2	5.69
7c1	F	F	Cl	H	1.23	5.91
7c2	F	F	NO_2_	H	4.14	5.38
7c3^a^	F	F	F	H	5.45	5.26
7c4	F	F	Br	H	2.26	5.64
7c5^a^	F	F	Br	CH_3_	1.45	5.83
7d1^a^	Cl	Cl	Cl	H	0.12	6.92
7d2	Cl	Cl	NO_2_	H	0.06	7.22
7d3	Cl	Cl	F	H	0.14	6.86
7d4	Cl	Cl	Br	H	0.15	6.82
7d5^a^	Cl	Cl	Br	CH_3_	0.2	6.69
7d6	Cl	Cl	Br	CO_2_C_2_H_5_	0.1	7

^a^Test compounds set.

**Table 2:** Uni-column statistics of the training and test sets for QSAR models

**Data set**	**Column name**	**Average**	**Max.**	**Min.**	**SD**	**Sum**
2D-QSAR						
Training	pEC_50_(μM)	5.9092	7.2300	4.5800	0.9019	70.9100
Test	pEC_50_(μM)	5.6940	6.9300	4.8600	0.6500	56.9400
3D-QSAR						
Training	pEC_50_(μM)	5.8417	7.2300	4.5800	0.7859	105.1500
Test	pEC_50_(μM)	5.6750	6.9300	4.8600	0.8967	22.7000

The maximum and minimum value in training and test set were compared in a way that:

1 The maximum value of pEC_50_ of test set should be less than or equal to maximum value of pEC_50_ of training set.

2 The minimum value of pEC_50_ of test set should be higher than or equal to minimum value of pEC_50_ of training set.

This observation showed that test set was interpolative and derived within the minimum–maximum range of training set. The mean and standard deviation of pEC_50_ values of sets of training and test provide insights to the relative difference of mean and point density distribution (along mean) of the two sets. *In vitro* effective concentrations (EC_50_) of the molecules were converted into corresponding pEC_50_ values (Table 1) and used as dependent variables in 2D QSAR calculations.

#### Descriptor calculation

The basis of energy minimization is that the drug binds to effectors/receptors in the most stable form, i.e., the minimum energy form. 2D-QSAR study requires the calculation of molecular descriptors. A large number of theoretical 2D individual descriptors such as Mol. Wt., Volume, XlogP, smr; physiochemical such as Estate Numbers, Estate contributions, Polar Surface Area, Element Count, Dipole moment, Hydrophobicity XlogpA, Hydrophobicity SlogpA; topological such as T_2_Cl_6, T_C_Cl_6, T_T_S_7, T_T_Cl_7 type have been computed for these geometrically optimized structures from the chemical structures of the compounds referred to above with a view to develop structure–activity relationship of 1,2,3thiadiazole thioacetanilides derivatives against the HIV. A total of 938 descriptors were calculated by QSARPlus module within VLife Sciences Molecular Design Suite. The descriptors having the same value or almost same value or highly correlated with other descriptors were removed initially, as they do not contribute to the QSAR. The reduced set of descriptors was then treated by Forward Stepwise Variable Selection for further reduction of non-significant descriptors and finally the optimum models with four significant descriptors were considered in our 2D-QSAR analysis.

#### Statistical analysis

The 2D-QSAR model was generated by Multiple Linear Regression (MLRmethod by using V-Life Molecular Design Suite (MDS). It relates the dependent variable **ŷ** (biological activity) to a number of independent variables *x*_*i*_ (molecular descriptor) by using linear equations. This method of regression estimates the values of the regression coefficients by applying least square curve fitting method. MLR is the traditional and standard approach for multivariate data analysis. Multivariate analysis is the analysis of multidimensional data metrices by using statistical methods. Such data metrices can involve dependent and/or independent variables. For getting reliable results, parameters were set such that the regression equation should generate number of independent variables (descriptors) 5 times less than that of compounds or molecules.

The multiple regression equation takes the form as mentioned in Equation(1)

(1) $$\hat{y}=b_{\text{o}}+b_{1}x_{1}+b_{2}x_{2}+b_{\text{p}}x_{\text{p}}$$

where *ŷ* = calculated dependent variable (biological activity pEC_50_)the *b*_1_ to *b*_*p*_ are regression coefficients [contribution of respective descriptors that is *x*_1_ to *x*_3_, *x*_1_ to *x*_*p*_ are independent variables (descriptors) and *b*_o_ is a regression constant or intercept].

The program computes the best model on the basis of squared correlation coefficient *r*^2^, crossed validated *q*^2^, which is relative measure of quality of fit, Fischer’s value *F*-test which represents *F*-ratio between the variance of calculated and observed activity and pred_*r*^2^. The calculated value of *F*-test when compared with tabulated value of *F*-test shows the level of statistical significance (99.99%) of the QSAR model. The low standard error of pred_*r*^2^ se, *q*^2^_se, and *r*^2^_se shows absolute quality of fitness of the model. The generated QSAR model was validated for predictive ability inside the model by using cross validation (leave-one-out—LOO) for *q*^2^ and external validation, which is more robust alternative method by dividing the data into training set & test set and calculating pred_*r*^2^. The high pred_*r*^2^ and low pred_*r*^2^ se were showed high predictive ability of the model.

**Figure 1:**
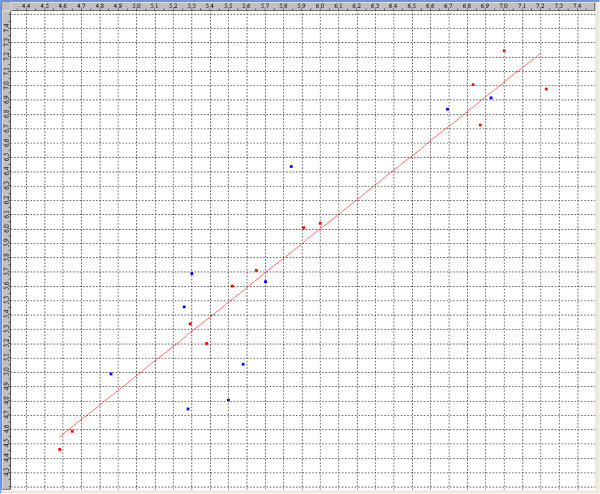
Comparison of observed activity versus predicted activity for training set & test set compounds according to 2D-QSAR model.

**Figure 2:**
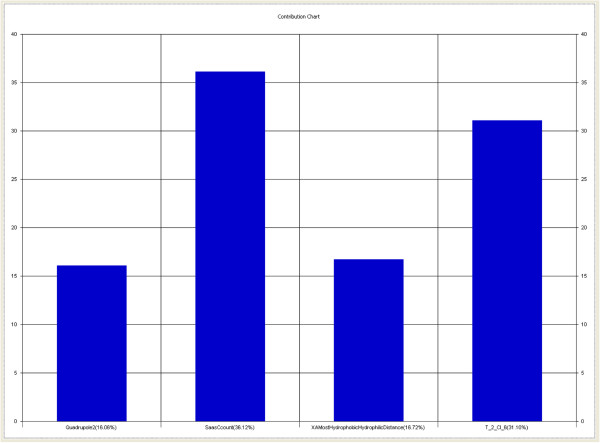
Contribution of descriptors for biological activity developed using MLR equation.

The statistical significance of selected 2D-QSAR model was further supported by the ‘fitness plot’ obtained, this is a plot of observed versus predicted activity of training and test set compounds and provides an idea about how well the model was trained & how well it predicts the activity of the external test set (Figure 1). The contribution chart for the significant model is presented in Figure 2, which gives the percentage contribution of the descriptors used in deriving the model.

### 3D-QSAR studies

#### Data set and molecular modeling for 3D-QSAR

The total set of compounds was divided into a training set (18 compounds) for generating 3D-QSAR models and a test set (4 compounds) for validating the quality of the models. Optimal training and test set were generated using the sphere exclusion (SE) algorithm. The SE method was adopted for division of training and test data set comprising of 18 and 4 molecules, respectively, with dissimilarity value of 13.4 where the dissimilarity value gives the SE radius. The SE method employs the following algorithm: (i) select a point and include it in the training set; (ii) build a sphere with radius *R* with a center in this point; (iii) include all points within the sphere, except for the center, in the test set; (iv) discard all points in the sphere from the initial set; (v) if no points are left, stop, otherwise go to step (i). The most active compound in the dataset is selected as the starting point for building a sphere. Four compounds, namely, 7d1, 7c3, 7a5, and 7c4, were used as test set while the remaining molecules were used as the training set (Table 3). The uni-column statistics of the training and test sets are reported in Table 2.

**Table 3:** Descriptors used in 2D & 3D-QSAR models with values

**ID**	**2D-QSAR descriptors**				**3D-QSAR descriptors**	
	**Saas C count**	**Quadrupole2**	**T_2_Cl_6**	**XA most hydrophobic hydrophilic distance**	**H_171**	**E_587**
7a1	5	16.12701	0	4.951941	0.337546	0.088079
7a4	5	22.34026	0	5.241149	0.340001	0.109635
7a5^a,b^	6	16.34572	0	4.63805	0.353856	0.057851
7a6^a^	6	3.079616	0	4.141958	0.342394	0.136266
7a7	6	−26.8322	0	7.462867	0.334305	0.117725
7b1^a^	6	10.24981	0	5.100435	0.350985	0.049167
7b2	6	30.74947	0	8.220488	0.332967	0.027609
7b4^a^	6	−38.4946	0	5.790629	0.345013	0.141681
7b5^a^	7	12.74566	0	5.113997	0.369819	−0.030558
7b6	7	−41.4985	0	6.625907	0.358049	0.154737
7b7^a^	7	−17.1541	0	5.913619	0.345649	0.196553
7c1	7	−13.5687	0	7.263344	0.368474	−0.008455
7c2	7	−46.9851	0	5.258711	0.329492	−0.064939
7c3^a,b^	7	−13.251	0	5.105986	0.335960	−0.034363
7c4^b^	7	−10.3413	0	5.992268	0.369205	0.042498
7c5^a^	8	−5.89083	0	6.29995	0.398119	0.028883
7d1^a,b^	7	−6.89132	2	6.834099	0.463411	0.019026
7d2	7	18.5915	2	6.196837	0.428789	−0.072705
7d3	7	−9.69926	2	6.197515	0.429396	−0.019153
7d4	7	36.29885	2	5.712653	0.437677	−0.026282
7d5^a^	8	−24.2613	2	4.75547	0.497335	−0.003169
7d6	8	−12.1663	2	5.933494	0.478959	−0.006141

^a^The compounds considered in the test set in 2D QSAR.

^b^The compounds considered in the test set in 3D QSAR.

#### Alignment procedure

Molecular alignment is a crucial step in 3D-QSAR study to obtain meaningful results. This method is based on moving of molecules in 3D space, which is related to the conformational flexibility of molecules. Energy minimized and geometry optimized structure of molecules were aligned by the template based. In general, geometric similarity should exist between the modeled structures and the bioactive conformation for 3D-QSAR. The spatial alignment of compounds under study is thus one of the most sensitive and determining factor in obtaining a reliable model. The alignments define the putative pharmacophore for the series of ligands. Alignment of all 22 compounds was done using the template-based alignment by using the most active molecule as reference and 1,2,3-thiadiazole thioacetanilides as a template in MDS; the aligned structures were used for the study. In the template-based alignment, a template structure was defined and used as a basis for alignment of a set of molecules. These aligned conformations were used to generate the predictive QSAR model. Multiple conformation of each molecule was generated using Monte Carlo conformation search method. It is a random search method for finding the conformations of molecules which uses the metropolis condition to accept or discard generated conformers [14].

#### Descriptors calculation

Using Tripos force field [15] and Gasteiger and Marsili charge type [16] electrostatic, steric and hydrophobic field descriptors were calculated. The dielectric constant was set to 1.0, considering the distance-dependent dielectric function probe setting was carbon atom with charge 1.0. This resulted in calculation of 5,040 field descriptors (1,680 for each electrostatic, steric, and hydrophobic) for all the compounds in separate columns. QSAR analysis was performed after removal of all the invariable columns, as they do not contribute to the QSAR.

3D-QSAR studies were carried out by kNN method using Forward Stepwise Variable Selection as variable selection method. The kNN methodology relies on a simple distance learning approach whereby an unknown member is classified according to the majority of its kNNsin the training set. The nearness is measured by an appropriate distance metrics (e.g., a molecular similarity measure calculated using field interactions of molecular structures). The standard kNN MFA method was implemented simply as follows

1. Calculated distances between an unknown object (*u*) & all the objects in the training set

2. Select *k* objects from the training set most similar to object *u*, according to the calculated distances; and

3. Classify object *u* with the group to which the majority of the *k* objects belong. An optimal *k* value is selected by optimization through the classification of a test set of samples or by leave-one out cross-validation.

The variables and optimal *k* values were chosen using stepwise variable selection method. This method employs a stepwise variable selection procedure combined with kNN to optimize.

1. The number of nearest neighbors (*k*) and

2. The selection of variables from the original pool.

The step-by-step search procedure begins by developing a trial model with a single independent variable and adds independent variables, one step at a time, examining the fit of the model at each step (using weighted kNN cross-validation procedure). The method continues until there are no more significant variables remaining outside the model. Once the training and test sets are generating, kNN methodology is applied to the descriptors generated over the grid. The steric, electrostatic, and hydrophobic energies are computed at the lattice points of the grid using a methyl probe of charge +1. These interaction energy values are considered for relationship generation and utilized as descriptors to decide the nearness between molecules.

*k*-Nearest neighbor molecular field analysis (kNN-MFA) models were developed using the Forward Stepwise Variable Selection method with cross-correlation limit set of 1.0 and the term selection criteria as *r*^2^. *F*-test ‘in’ was set to 4.0. As some additional parameters, variance cutoff was set at 0.000 kcal/molÅ and scaling to none; additionally, kNN parameter setting was done within the range of 2–5 and the prediction method was selected as the distance-based weighted average.

#### Crossvalidation

The standard LOO procedure was implemented that is a molecule in the training set was eliminated and its biological activity was predicted as the weighted average activity of the *k* most similar molecules (Equation 2)

(2) $${\hat{y}}_{i}=\sum w_{i}y_{i}$$

The similarities were evaluated as the inverse of Euclidean distance between molecules (Equation 3) using only the subset of descriptors

(3) $${\text{v}}_{\text{n}}$$

(4) $$d_{i,j}={\left[{}^{}\sum \left(X_{i,k}–X^{2}{}_{j,k}\right)\right]}^{1/2}$$

(5) k=1

This step was repeated until every molecule in the training set has been eliminated and its activity predicted once. The cross-validation *r*^2^ (*q*^2^) value was calculated using Equation(4) where *y*_*i*_ and *ŷ*_*i*_ are the actual and the predicted activities of the *i*th molecule, respectively, and *y*_mean_ is the mean of observed activity of all molecules in training set. Both summations are over all molecules in the training set.

(6) $$\sum {\left(y_{i}–{\hat{y}}_{i}\right)}^{2}$$

(7) $$q^{2}=1$$

(8) $$\sum {\left(y_{i}–y_{\text{mean}}\right)}^{2}$$

Since the calculation of the pairwise molecular similarities and hence the prediction was based upon current training set, the *q*^2^ value obtained (0.89) is the indicative power of the current kNN-MFA model. The above steps were repeated for *k* = 1 to 18 (upper limit of *k* is the total number of molecules in the dataset). The optimum value of *k* for our training set was found to be 2 (this value led to the highest *q*^2^ value, 0.89).

#### External validation

The external validation that is predicted *r*^2^ (pred_*r*^2^) value was calculated using the following equation where *y*_*i*_ and *ŷ*_*i*_ are the actual and the predicted activities of the *i*th molecule in the test set, respectively, and *y*_mean_ is the observed activity of all molecules in training set.

(9) $$\sum {\left(y_{i}-{\hat{y}}_{i}\right)}^{2}$$

(10) $$\text{pred}\_r^{2}=1$$

(11) $$\sum {\left(y_{i}-y_{\text{mean}}\right)}^{2}$$

Both the summations are over all the molecules in the test set. The pred_*r*^2^ value is indicative of all the predictive power of the current kNN-MFA model for external test set. The pred_*r*^2^ value of our 3D model was found to be 0.97.

#### Randomization test

To evaluate the statistical significance of the QSAR model for an actual dataset, we have employed a one-tail hypothesis testing. The robustness of the QSAR models for experimental training sets was examined by comparing these models to those derived for random datasets. Random sets were generated by rearranging biological activities of the training set molecules. The significance of the models hence obtained was derived on calculated *Z* score [17,18].

#### Evaluation of model

Developed quantitative model was evaluated using following statistical measures: *N*, number of observations (molecules) in the training set; Number of nearest neighbors, number of *k*-nearest neighbor in the model; *q*^2^, cross-validated *r*^2^ (by LOO) which is relative measure of quality of fit; pred_*r*^2^, *r*^2^ for external test set; *q*^2^_se, standard error of cross-validation and pred_*r*^2^ se, standard error of external test set prediction.

The low standard error of pred_*r*^2^ se and *q*^2^_se shows absolute quality of fitness of the model. The high pred_*r*^2^ and low pred_*r*^2^ se were showed high predictive ability of the model.

The *q*^2^ and pred*_r*^2^ values were used as deciding factors in selecting the optimal models.

### Docking studies

These studies helped to sort out the designed compounds with good binding affinity against RT enzyme. The molecular docking tool, GLIDE (Schrodinger Inc., USA) software, was used for studying binding modes of the designed compounds in to the binding pocket of RT enzyme. To further understand the binding mode between 1,2,3-thiadiazole thioacetanilides analogs and HIV-1 NNRT, these compounds were docked to the core domain of NNRT. Fine 3D structure with a resolution of 2.65 Å of NNRT was retrieved from the Protein Data Bank (*PDB ID: 1KLM*). Before docking, the protein is prepared by using the protein preparation wizard, removing the water molecules and cofactors from the proteins, optimizing hydrogen bonding and deleting the ligand present in crystal structure.

## Results and discussion

### 2D-QSAR

The various 2D-QSAR models were developed using MLR method. 2D-QSAR equations were selected by optimizing the statistical results generated along with variation of the descriptors in these models. The fitness/pattern plots were also generated for evaluating the dependence of the biological activity on various different types of the descriptors. The frequency of use of a particular descriptor in the population of equations indicated the relevant contributions of the descriptors.

The best regression equation obtained is represented in Equation(6):

pEC_50_ = 0.607826 SaasCcount + 0.00878695 Quadrupole2 + 0.478468 T_2_Cl_6 + 0.257279 XAMostHydrophobicHydrophilicDistance + 0.00652846 (6)

The equation explains 97% (*r*^2^ = 0.97) of the total variance in the training set. It also has an internal (*q*^2^) and external (pred_*r*^2^) predictive ability of approximately 94 and 62%, respectively. The *F*-test = 60.77 shows the statistical significance of 99.99% of the model which means that probability of failure of the model is 1 in 10,000. In addition, the randomization test shows confidence of approximately 99.9% that the generated model is not random and hence it is chosen as the QSAR model (Table 4).

**Table 4:** Observed, predicted activity, and residual values of statistically significantly models obtained by MLR & kNN-MFA

**ID**	**Observed activity pEC**_**50**_	**Predicted activity pEC**_**50**_**by MLR**	**Residual values of pEC**_**50**_**by MLR**	**Predicted activity of pEC**_**50**_**by kNN-MFA**	**Residual values of pEC**_**50**_**by kNN-MFA**
7a1	4.58	4.46	0.12	4.97	−0.39
7a4	4.64	4.59	0.5	4.94	−0.30
7a5^a,b^	4.86	4.99	0.13	5.08	−0.15
7a6^a^	5.28	4.74	0.46	5.39	−0.11
7a7	5.29	5.33	0.4	4.96	0.33
7b1^a^	5.58	5.05	0.53	5.30	0.28
7b2	6	6.038	−0.38	5.74	0.26
7b4^a^	5.49	4.80	−0.31	5.40	0.9
7b5^a^	5.29	5.68	−0.39	5.65	−0.36
7b6	5.52	5.60	−0.8	5.39	0.13
7b7^a^	5.69	5.63	0.6	5.51	0.18
7c1	5.91	6.01	−0.10	5.57	0.34
7c2	5.38	5.20	0.18	5.60	−0.22
7c3^a,b^	5.26	5.45	0.19	5.34	−0.8
7c4^b^	5.64	5.71	−0.7	5.70	−0.6
7c5^a^	5.83	6.43	0.40	5.75	0.8
7d1^a,b^	6.92	6.92	0	6.84	0.8
7d2	7.22	6.97	0.25	6.85	0.37
7d3	6.86	6.73	0.13	6.91	−0.5
7d4	6.82	7	0.18	6.93	−0.11
7d5^a^	6.69	6.83	−0.14	6.92	−0.23
7d6	7	7.24	−0.24	6.76	0.24

^a^The compounds considered in the test set in 2D QSAR.

^b^The compounds considered in the test set in 3D QSAR.

The plot of observed versus predicted activity provides an idea about how well the model was trained and how well it predicts the activity of the external test set. From the plots it can be seen that the model is able to predict the activity of training set quite well (all points are close to regression line) as well as external test set up to 62% (only few points are relatively apart from the regression line) providing confidence in predictive ability of the model (Figure 1).

#### Interpretation of result

It is apparent from Equation (6) that the descriptor SaasCcount plays a pivotal role in determining activity. This descriptor (SaasCcount) signifies the total number of carbon connected with one single bond along with two aromatic bonds. Positive contribution of this descriptor revealed the increase of anti-HIV activity of 1,2,3-thiadiazole thioacetanilides with presence of more number of carbon connected with single bond along with two aromatic bonds (Figure 2). The positive coefficient of SaasCcount (36%) showed that increase in the values of this descriptor is beneficial for anti-HIV activity (like in compounds 7c1, 7c5, 7d1, 7d2, 7d3, 7d4, 7d5, and 7d6). The next most important factor governing variation in the activity is T_2_Cl_6 (approximately 31%) and is directly proportional to the anti-HIV activity. The descriptor T_2_Cl_6 indicates that the count of number of double-bonded atoms (i.e., any double-bonded atoms, T_2) separated from chlorine atom by six bonds. The descriptor T_2_Cl_6 reveals the importance of chlorine atom at *R*^2^ position to be favorable for the activity. The positive coefficient of T_2_Cl_6 (approximately 31%) showed that increase in the values of this descriptor is beneficial for anti-HIV activity (like in compounds 7d1, 7d2, 7d3, 7d4, 7d5, and 7d6). As a positive contributing descriptor, XAMostHydrophobicHydrophilicDistance [signifies distance between most hydrophobic and hydrophilic point on the Vender Waals (vdW) surface] is also a physico-chemical descriptor influencing activity variation and is directly proportional to activity. The positive coefficient of XAMostHydrophobicHydrophilicDistance (17%) showed that increase in the values of this descriptor is beneficial for anti-HIV activity (like in compounds 7b2, 7c1, 7c5, 7d1, 7d2, and 7d3). The last descriptor is Quadrupole2 which shows the importance of magnitude. This descriptor is an individual descriptor that signifies magnitude of second tensor of quadrupole moments positively contributes to the biological activity (16%). The positive coefficient of Quadrupole2 showed that increase in the values of this descriptor is beneficial for anti-HIV activity (like in compounds 7b2, 7d2, and 7d4).

### 3D-QSAR

For 3D-QSAR, a kNN-MFA of 1,2,3-thiadiazole thioacetanilides derivatives with reported activities against HIV was prepared. Several 3D-QSAR models were generated using stepwise variable selection method resulted several statistically significant models, of which the corresponding best model is reported herein. 3D-QSAR model was selected based on the value of statistical parameters and the best kNN-MFA 3D-QSAR model with 18 training set compounds have a *q*^2^ = 0.89 and pred_*r*^2^ = 0.97.

**Figure 3:**
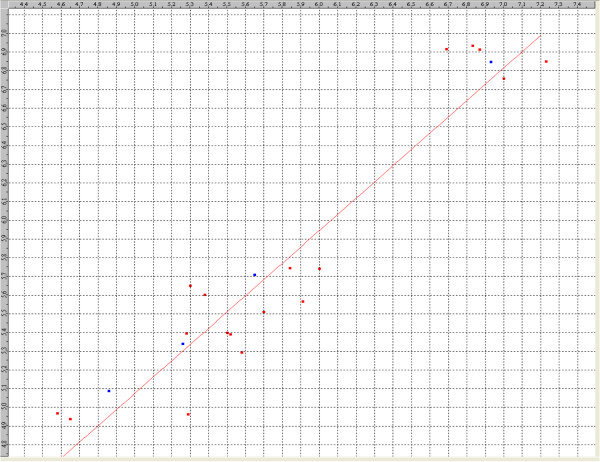
Comparison of observed activity versus predicted activity for training set and test set compounds according to 3D-QSAR model.

The kNN-MFA QSAR method explores formally the active analog approach which implies that compounds display similar profiles of pharmacological activities. In this method, the activity of each compound is predicted as average activity of *k* most chemically similar compounds from the dataset. The predictive ability of this Forward Stepwise Variable Selection kNN-MFA model was evaluated by predicting the biological activities of the test set molecules. Residuals values obtained by subtraction of predicted activities from biological activities were found near to zero. Therefore, it was concluded that the resultant QSAR model have good predictive ability. The actual, predicted activities,and residuals of both training and test sets molecules are given in Table 5. The plots of observed versus predicted activity of both training and test sets molecules helped in cross-validation of kNN-MFA QSAR model are depicted in Figure 3.

**Table 5:** Statistical results of 2D QSAR equation generated by MLR method and 3D QSAR models generated by forward stepwise variable selection kNN MFA method for 1,2,3-thiadiazole thioacetanilides derivatives

**Serial number**	**Statistical parameter**	**Results**	
		**2D QSAR by MLR**	**3D QSAR by kNN**
1	*r*^2^	0.97	–
2	*r*___^2^se	0.19	–
3	*q*^2^	0.94	0.89
4	*q*___^2^se	0.29	0.26
5	pred_*r*^2^	0.62	0.97
6	pred_*r*^2^se	0.43	0.15
7	*F*-test	60.77	–
8	*N*	12	18
9	Nearestneighbor	–	2
10	Degree of freedom	7	15
11	Contributing descriptors	SaasCcount (36.12%) Quadrupole2 (16.06%)T_2_Cl_6 (16.72%) XAMostHydrophobicHydrophilicDistance(31.10%)	H_171 (0.429396, 0.437677) E_587 (−0.026282, –0.019153)

The model selection criterion is the value of *q*^2^, the internal predictive ability of the model and that of pred_*r*^2^, the ability of the model to predict the activity of external test set. For anti-HIV activity, selected model was found to be statistically most significant, especially with respect to the internal predictive ability (*q*^2^ = 0.89) of the model. As the cross-validated correlation coefficient (*q*^2^) is used as a measure of reliability of prediction, the correlation coefficient suggests that our model is reliable and accurate. A dataset of 4 compounds was selected as the test set from the original data of 22 compounds for the validation experiments. The predicted versus the experimental selectivity values for the training and test sets are depicted in Figure 3. The value of pred_*r*^2^ was obtained for the test set and gave better results, with a value of 0.97, which means 97% predictive power for the external test set. Thus, our model displays good predictivity in regular crossvalidation (Table 4).

**Figure 4:**
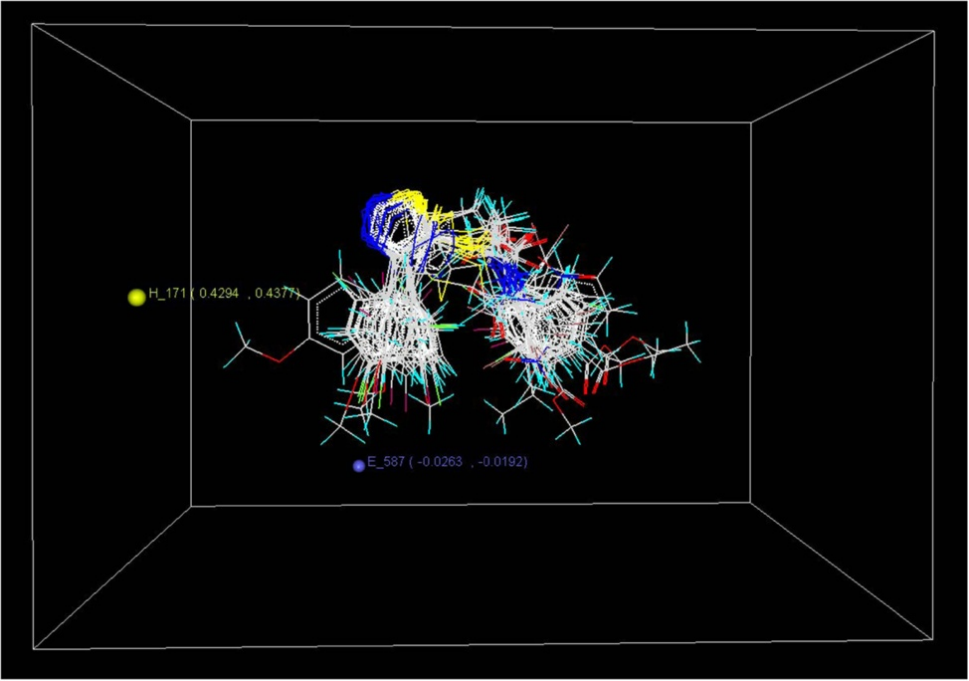
Stereo view of the molecular rectangular field grid around the superposed molecular units of 1,2,3-thiadiazole thioacetanilides series of compounds using SW-kNN MFA method.

In 3D-QSAR studies, 3D data points generated 1,2,3-thiadiazole thioacetanilides pharmacophore were used to optimize the electrostatic and hydrophobic requirements of the 1,2,3-thiadiazole thioacetanilides nucleus for the anti-HIV activity. The range of property values in the generated data points helped for the design of NCEs. These ranges were based on the variation of the field values at the chosen points using the most active molecule and its nearest neighbor set. The points generated in SW-kNN MFA 3D-QSAR model are H_171 (0.429396, 0.437677) and E_587 (−0.026282, –0.019153) that is hydrophobic and electrostatic interaction field at lattice points 171 and 587, respectively (Figure 4). These points suggested the significance and requirement of hydrophobic and electrostatic properties as mentioned in the ranges in parenthesis for SAR and maximum biological activities of 1,2,3-thiadiazole thioacetanilides analogues.

From 3D-QSAR model, it is observed that electrostatic descriptor like E_587 (−0.026282, –0.019153) with negative values is near from *R*_2_ position of the 1,2,3-thiadiazole thioacetanilides ring. This indicates that electronegative groups are favorable on this site and presence of electronegative groups increases the activity of 1,2,3-thiadiazole thioacetanilides analog. Most potent compounds (compounds 7d1, 7d2, 7d3, 7d4, 7d5, and 7d6) with higher activity having electronegative substitution (Cl) at the *R*_2_ position of the 1,2,3-thiadiazole thioacetanilides ring strongly support the above statement. The negative values of electrostatic descriptors suggested the requirement of electronegative group like NO_2_, SO_2_R, CN, SO_2_Ar, COOH, F, Cl, Br, I, COOR, OR, OH at the position of generated data point E_587 (−0.026282, –0.019153) around 1,2,3-thiadiazole thioacetanilides pharmacophore for maximum activity.

The presence of hydrophobic descriptor H_171 with positive values is also near from the *R*_1_ position of the 1,2,3-thiadiazole thioacetanilides ring which indicates that more hydrophobic substituents are favorable on this site and presence of more hydrophobic substituents increases the anti-HIV activity of 1,2,3-thiadiazole thioacetanilides compounds. Therefore, more hydrophobic substituents such as –C_6_H_11_, –C_6_H_5_, –C(CH_3_)_3_, –CF_3,_–CH_3_, etc., were preferred at the position of generated data point H_171 around 1,2,3-thiadiazole thioacetanilides pharmacophore.

### Molecular docking studies

The docking score in terms of G-Score (GLIDE Score), Emodel, and other results of docking studies of designed compounds of TTA series are presented in Table 6.

**Table 6:** Results of molecular docking

**Serial number**	**ID**	**G-Score**	**LipophilicEvd W**	**H-bond**	**Electro**
1	7d1	−12.29	−7.22	−0.65	0.01
2	Efavirenz	−11.61	−5.59	−0.7	−0.21
3	7c5	−11.29	−6.7	−0.7	−0.36
4	7c4	−11.17	−6.53	−0.7	−0.35
5	7d3	−11.09	−7.6	−0.51	−0.11
6	7d4	−10.97	−7.99	0	−0.11
7	7c3	−10.95	−6.51	−0.7	−0.2
8	7d6	−10.9	−6.79	−0.7	−0.37
9	7c1	−10.83	−6.21	−0.7	−0.33
10	7b4	−10.81	−6.13	−0.68	−0.21
11	Zidovudine	−10.81	−3.68	−2.17	−0.47
12	7b1	−10.75	−6.03	−0.7	−0.25
13	7b2	−10.75	−6.77	−0.57	0.12
14	7d5	−10.6	−6.93	−0.7	−0.38
15	7b5	−10.46	−6.17	−0.6	−0.2
16	7b6	−10.38	−6.65	−0.7	−0.36
17	7a7	−10.33	−6.74	−0.7	−0.3
18	7b7	−10.15	−6.58	−0.7	−0.25
19	7a1	−10.15	−5.46	−0.61	−0.19
20	7c2	−10.13	−6.27	−0.67	−0.16
21	7a5	−10.05	−6.67	−0.7	−0.44
23	7a4	−9.98	−6.52	−0.7	−0.37
24	7a6	−9.92	−7.37	0	−0.11
25	Delavirdine	−9.89	−7.63	−1.27	−0.74
26	7d2	−9.47	−6.98	0	−0.05
27	Nevirapine	−6.59	−4.88	0	−0.1

#### *G-score*

The scoring function of GLIDE docking program is presented in the G-score form. The G-score indicates the binding affinity of the new compound to the receptor/enzyme.

The G-score of the standard compound zidovudine and efavirenz was found to be −10 and −11.61, respectively. The G-score of the series compounds 7c5, 7c4, 7d3, 7d4, 7c3, 7d6, 7c1, 7b4 was found to be more than zidovudine,and the 7d1 having highest G-score −12.29 which is more than efavirenz. The close analysis of these results suggests that the new series have comparable G-score with the standard compounds.

#### H-bond

**Figure 5:**
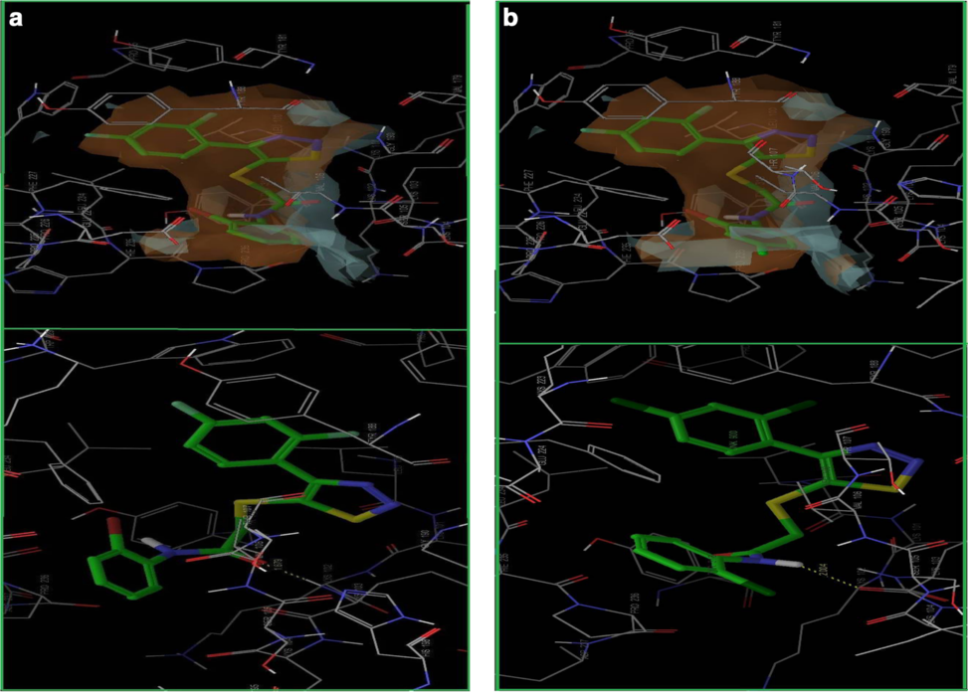
**Binding interaction of compounds with NNRT receptors.** (**a**) Compounds 7c4 interacting with NNRT receptor protein of PDB *1KLM*. (**b**) Compounds 7c5 interacting with NNRT receptor protein of PDB *1KLM*. Dotted yellow bond showing H-bond interactions with binding site residues. Grey surface showing lipophilic interaction and brown color showing electrostatic interactions.

H-bond is one of the most widely used parameter for the evaluation of the docking results, as it is an influential parameter in the activity of the drug compound. The number of H-bond interactions in the standard compounds was compared with that of new series. The number of compounds shows a good H-bond interaction. Figure 5 has shown that compounds 7c5 and 7c4 show good H-bonding interaction with LYS102, LYS104, and LYS103 residues of NNRT, respectively.

#### vdWinteraction

The contacts are represented in the form of vdWinteraction.

• Good vdW interactions.

• Bad vdW interactions.

• Ugly vdW interactions.

The developed molecules show good number of vdW interaction. The molecule 7d1 shows highest lipophilic character −7.22 which is higher than standard compound efavirenz which show −5.59 only. The molecules 7c5,7c4, 7d3, 7d4, 7c3, 7d6, 7c1, 7b4 having optimum lipophilicity which is greater than standard compound zidovudine.

## Conclusions

Our aim in this study was to biologically evaluate a series of analogs of 1,2,3-thiadiazole thioacetanilides by modifying systematically the molecule, in order to explore the SAR of these derivatives. In order to determine the better structural characteristics that were able to improve the anti-HIV-1 activity of this class of NNRTIs and to investigate the effects of different chemical modifications on the RT inhibition, an extensive SAR was examined by varying the nature and the position of the substituents both on the basic moiety. Positive values in hydrophobic field descriptor indicated the requirement of positive hydrophobic potential for enhancing the biological activity of 1,2,3-thiadiazole thioacetanilides analog. Series compounds 7c5, 7c4, 7d3, 7d4, 7c3, 7d6, 7c1, 7b4 showing good binding affinity as compared to standard drugs which revealed that the nature of the substituent and substitution pattern on the basic ring may have a considerable impact on the NNRTIs activity of the synthesized compounds.

## Competing interests

The authors declare that they have no competing interests.
